# Analysis of the Subdivision Errors of Photoelectric Angle Encoders and Improvement of the Tracking Precision of a Telescope Control System

**DOI:** 10.3390/s18092998

**Published:** 2018-09-07

**Authors:** Jiawei Yu, Qiang Wang, Guozhong Zhou, Dong He, Yunxia Xia, Xiang Liu, Wenyi Lv, Yongmei Huang

**Affiliations:** 1 Institute of Optics and Electronics, Chinese Academy of Sciences, No. 1 Guangdian Road, Chengdu 610209, China; Yujiawei11@126.com (J.Y.); qiangwang@ioe.ac.cn (Q.W.); Zgz_ioe@sina.cn (G.Z.); hedong@ioe.ac.cn (D.H.); xyxhaha@163.com (Y.X.); lx061990@163.com (X.L.); lvwy@mail.ustc.edu.cn (W.L.); 2 Key Laboratory of Optical Engineering, Chinese Academy of Sciences, Chengdu 610209, China; 3 University of Chinese Academy of Sciences, Beijing 100049, China

**Keywords:** telescope control systems, photoelectric angle encoders, tracking precision, subdivision errors, amplitude error, real-time compensation

## Abstract

Photoelectric angle encoders, working as position sensors, have a great influence on the accuracy and stability of telescope control systems (TCS). In order to improve the tracking precision of TCS, a method based on subdivision error compensation for photoelectric angle encoders is proposed. First, a mathematical analysis of six types of subdivision errors (DC error, phase error, amplitude error, harmonic error, noise error, and quantization error) is presented, which is different from the previously used analysis based on the Lissajous figure method. In fact, we believe that a mathematical method is more efficient than the figure method for the expression of subdivision errors. Then, the distribution law and period length of each subdivision error are analyzed. Finally, an error compensation algorithm is presented. In a real TCS, the elevation jittering phenomenon occurs, which indicates that compensating for the amplitude error is necessary. A feed-forward loop is then introduced into the TCS, which is position loop- and velocity loop-closed, leading to a decrease of the tracking error by nearly 54.6%, from 2.31” to 1.05”, with a leading speed of 0.25°/s, and by 40.5%, from 3.01” to 1.79”, with a leading speed of 1°/s. This method can realize real-time compensation and improve the ability of TCS without any change of the hardware. In addition, independently of the environment and the kind of control strategy used, this method can also improve the tracking precision presumably because it compensates the measuring error inside the photoelectric angle encoder.

## 1. Introduction

With the development of free space optical communication, a higher tracking precision of telescope control systems (TCS), which usually display arc second-scale accuracy [1,2], is a necessity. The photoelectric angle encoder is a kind of angular sensor, which achieves high resolution by the subdivision of two-way orthogonal moire pattern (sine and cosine) signals from a fine code disk [3]. Obviously, high-precision angle encoders contribute to the high accuracy of TCS. However, the real photoelectric parameters are not well matched with the processing circuit, and the normal subdivision methods are based on standard sine and cosine signals, which causes subdivision errors [4].

Scientists are working to manufacture high-precision and high-resolution photoelectric angle encoders. St. Petersburg State Electrotechnical University developed an encoder error detection system with an accuracy of 0.1” at the speed of up to 10 Round Per Second (RPS), which can realize dynamic error detection [5]. Wan et al. from Changchun Institute of Optics, Fine Mechanical, and Physics, Chinese Academy of Sciences, designed a high-precision photoelectric angle encoder with a dual-reading system, which satisfies the technique requirements of spaceborne equipment [6]. However, all these high-resolution angle measurements depend on large code disks. It is a challenge for scientists to achieve high resolution with small code disks [7].

To compensate the subdivision error of the photoelectric angle encoders, whose code disks are small, Wang et al. proposed an error compensation method based on an improved Back Propagation (BP) neural network, which brought a 15% improvement in accuracy [8]. An experimental–theoretical method was proposed by E. V. Kaplya to identify and compensate static errors in the phase coordinates of the elements of an optical encoder and decreased the error of the encoder by 10.8% [9]. Yu’s team designed a 23-bit angle reference encoder by structure and circuit devising, whose subdivision error was then compensated by a Radical Basis Function (RBF) neural network. The resolution of the angle encoder was 0.15”, and, after compensating, the error of the encoder decreased from 1.3” to 0.6” [10]. An automatic compensation method for sine deviation, proposed by Gao and his cooperators, used particle swam optimization to identify the seven undefined parameters of the signal model and could reduce the subdivision error from 0.923” to 0.316” [11]. A method based on moire triangle subdivision error compensation, which was proposed by Sun, decreased the original error by 1/3 [12].

However, all the studies mentioned above have their shortcomings. For those based on neural or improved neural networks, their training sets are too big and need a long processing time before they can be implemented. Besides, when working in real systems, neural network-based methods need a considerable time to match the data input with the training sets. This is not practical for spaceborne equipment. For some other methods, the compensating strategies are difficult to adapt to changing working sites.

To improve the precision of photoelectric encoders, we designed a compensation method without changing the parameters of the hardware. First, we defined the position error of the encoder and identified the type of subdivision error corresponding to the main error. Then, we compensated the main error through the properties of the angle encoder. Finally, we improved the structure of the control system to further increase its precision. This compensation method required a short time and significantly increased the precision of the system. Most importantly, this method works in real time and can satisfy the requirements of spaceborne equipment. 

## 2. Mathematical Analysis on Subdivision Errors

Ideal grating fringe photoelectric signals are sine and cosine signals with equal amplitude, which can be expressed as follow [11]:

(1) $$u_{a}=A\sin\theta ,u_{b}=A\cos\theta ,$$

where A is the amplitude of the signal, and θ is the theoretic rotary angle of the signal; the angle θ can be deduced from:

(2) $$\theta =\arctan\frac{u_{a}}{u_{b}},$$

However, some factors influence the precision of subdivision, for example, the precision of the code disk, the precision of the axis, the quality of the photoelectric signals, the performance of the processing circuit, and so on. The real outputs are obtained by the moire fringe technique, which can be expressed as:

(3) $$u_{a}=A_{0}+A_{1}\sin(\theta +\varphi_{1})+\sum_{i=2}^{\infty }A_{i}\sin(i\theta +\varphi_{ia})+\delta_{a},$$

(4) $$u_{b}=B_{0}+B_{1}\sin(\theta +\varphi_{2})+\sum_{i=2}^{\infty }B_{i}\sin(i\theta +\varphi_{ib})+\delta_{b},$$

where the two signals consist of four parts.

*A*_0_ and *B*_0_ are DC components, which cause the DC subdivision error. *A*_1_ and *B*_1_ are the amplitudes of base waves, which cause the amplitude subdivision error. $\sum_{i=2}^{\infty }A_{i}\sin\left(i\theta +\varphi_{ia}\right)$ and $\sum_{i=2}^{\infty }B_{i}\sin\left(i\theta +\varphi_{ib}\right)$ are the sum of higher harmonics, which cause the harmonic subdivision error.

$\delta_{a}$ and $\delta_{b}$ are the electric noise, which causes the electric noise subdivision error. In addition, the analog-to-digital conversion (A/D) causes the quantization subdivision error. Also, the difference between the phases $\varphi_{1}$ and $\varphi_{2}$ causes the phase subdivision error [8].

Here, we define Δθ as the subdivision error, $\theta_{r}$ as the real rotary angle, and $\theta_{d}$ as the measured rotary angle. We have, then, the following equation:

(5) $$\Delta \theta =\theta_{r}-\theta_{d},$$

### 2.1. DC Subdivision Error Analysis

If there is only the DC subdivision error, we can introduce the DC components a,b into the expression of the subdivision error Δθ and then simplify it as shown:

(6) $$\Delta \theta =\theta_{r}-\arctan\frac{r\sin\theta_{r}+a}{r\cos\theta_{r}+b},$$

(7) $$\mathrm{so}:\tan\Delta \theta =\tan(\theta_{r}-\arctan\frac{\sin\theta_{r}+\frac{a}{r}}{\cos\theta_{r}+\frac{b}{r}}),$$

We can simplify the equation above:

(8) $$\tan\Delta \theta =\frac{b\sin\theta_{r}-a\cos\theta_{r}}{r+b\cos\theta_{r}+a\sin\theta_{r}},$$

Here, we define:

(9) $$\cos\varphi =\frac{b}{\sqrt{a^{2}+b^{2}}},\sin\varphi =\frac{a}{\sqrt{a^{2}+b^{2}}},$$

We can use zero shift of the angle (initial angle α) to calculate φ:

(10) $$\alpha =\arctan(\frac{a}{1+b}),$$

Then:

(11) $$\tan\Delta \theta =\frac{\sin(\theta_{r}-\varphi )}{\frac{r}{\sqrt{a^{2}+b^{2}}}+\cos(\theta_{r}-\varphi )},\varphi =\arctan\frac{a}{b},$$

Finally, we can get:

(12) $$\Delta \theta =\arctan\left(\frac{\sin(\theta_{r}-\varphi )}{\frac{r}{\sqrt{a^{2}+b^{2}}}+\cos(\theta_{r}-\varphi )}\right)=\arctan(\frac{\sin(\theta_{d}+\Delta \theta -\varphi )}{\frac{r}{\sqrt{a^{2}+b^{2}}}+\cos(\theta_{d}+\Delta \theta -\varphi )})$$

According to the equation above, we can use the measured rotary angle $\theta_{d}$ and α to get the DC subdivision error Δθ and compensate it.

**Figure 1:**
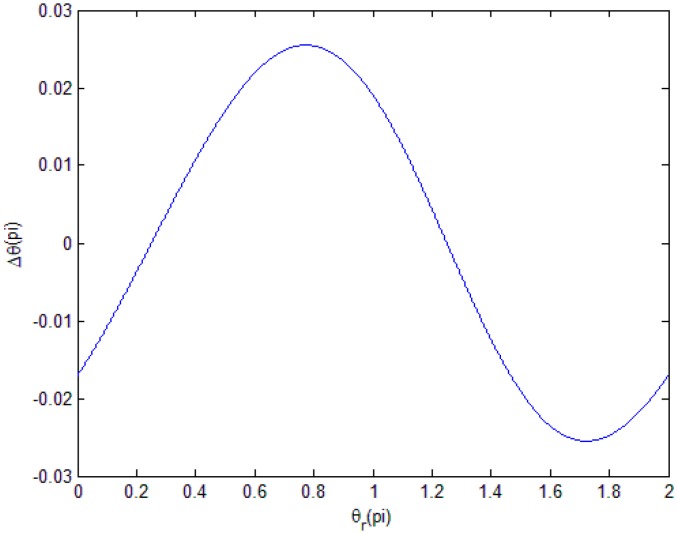
Distribution law of $\Delta \theta_{D}$ in one period of $\theta_{r}$.

Then, we can determine the change of Δθ when $\theta_{r}$ ranges from 0 to 2*π* and obtain the distribution law and period length of the DC subdivision error in Figure 1.

Here, we consider as an example the DC components a=0.2r,b=0.2r.

Each type of error has a special distribution law, which can help us to identify the main error. Here, we can see that, in one period of $\theta_{r}$, Δθ is a single period.

### 2.2. Magnitude Subdivision Error Analysis

If there is only the magnitude subdivision error, we can introduce the magnitude components dr into the expression of the subdivision error Δθ and then simplify it as follows:

(13) $$\Delta \theta =\theta_{r}-\arctan\frac{(r-dr)\sin\theta_{r}}{r\cos\theta_{r}},$$

(14) $$\mathrm{so}:\tan\Delta \theta =\frac{\tan\theta_{r}-\frac{(r-dr)\sin\theta_{r}}{r\cos\theta_{r}}}{1+\tan\theta_{r}\frac{(r-dr)\sin\theta_{r}}{r\cos\theta_{r}}},$$

We can finally simplify it:

(15) $$\Delta \theta =\arctan(\frac{\sin2\theta_{r}}{\cos2\theta_{r}+\frac{2r}{dr}-1})=\arctan(\frac{\sin(2\theta_{d}+2\Delta \theta )}{\cos(2\theta_{d}+2\Delta \theta )+\frac{2r}{dr}-1}),$$

According to the equation above, we can use the measured rotary angle $\theta_{d}$ and $\frac{dr}{r}$ to obtain the magnitude subdivision error Δθ and compensate it.

**Figure 2:**
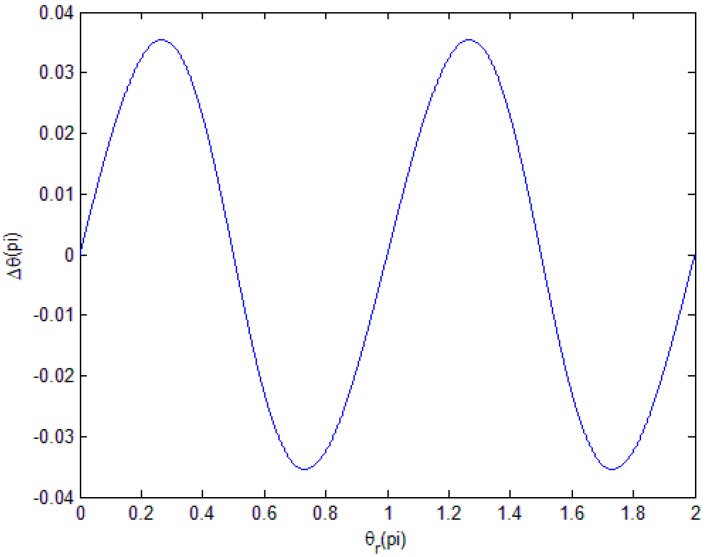
Distribution law of $\Delta \theta_{M}$ in one period of $\theta_{r}$.

Then, we can determine the change of Δθ when $\theta_{r}$ ranges from 0 to 2*π* and obtain the distribution law and the period length of the magnitude subdivision error in Figure 2.

Here, we consider the magnitude component dr=0.2r.

We can see that, in one period of $\theta_{r}$, Δθ is a dual period.

### 2.3. Phase Subdivision Error Analysis

If there is only the phase subdivision error, we can introduce the phase component $\varphi_{3}$ into the expression of the subdivision error Δθ and then simplify it as follows:

(16) $$\Delta \theta =\theta_{r}-\arctan\frac{r\sin(\theta_{r}+\varphi_{3})}{r\cos\theta_{r}},$$

(17) $$\mathrm{next}:\arctan(\frac{r\sin(\theta_{r}+\varphi_{3})}{r\cos\theta_{r}})=\theta_{r}-\Delta \theta =\theta_{d},$$

(18) $$\mathrm{so}:\Delta \theta =\arctan(\frac{\tan\theta_{d}}{\cos\varphi_{3}}-\tan\varphi_{3})-\theta_{d},$$

According to the equation above, we can use the measured rotary angle $\theta_{d}$ and $\varphi_{3}$ to get the phase subdivision error Δθ and compensate it.

**Figure 3:**
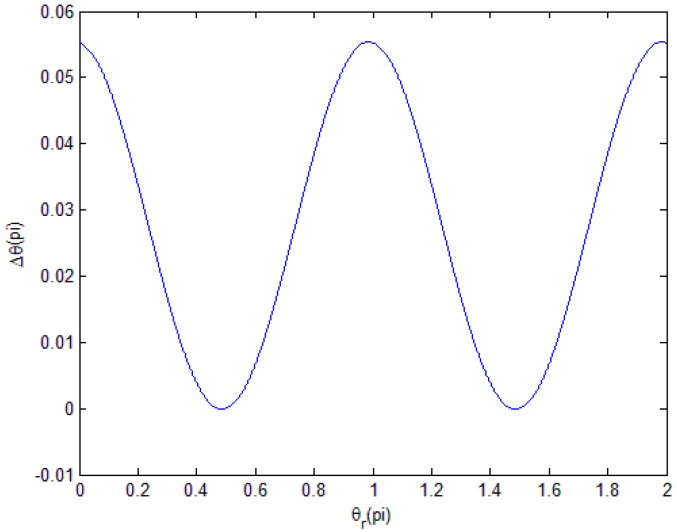
Distribution law of $\Delta \theta_{P}$ in one period of $\theta_{r}$.

Then, we can determine the change of Δθ when $\theta_{r}$ ranges from 0 to 2*π* and obtain the distribution law and period length of the phase subdivision error in Figure 3.

Here, we consider the phase components $\varphi_{3}=10{}^{\circ}$.

We can see that, in one period of $\theta_{r}$, Δθ is a dual period.

### 2.4. Harmonic Subdivision Error Analysis

If only the harmonic subdivision error is present, we can introduce the harmonic component $N\theta_{r}$ into the expression of the subdivision error Δθ and then simplify it as follows:

(19) $$\Delta \theta =\theta_{r}-\arctan\frac{A_{1}\sin\theta_{r}+\sum_{N=2}^{\infty }A_{N}\sin(N\theta_{r})}{A_{1}\cos\theta_{r}+\sum_{N=2}^{\infty }A_{N}\cos(N\theta_{r})},$$

(20) $$\mathrm{next}:\tan\Delta \theta =\frac{\tan\theta_{r}-\frac{A_{1}\sin\theta_{r}+\sum_{N=2}^{\infty }A_{N}\sin(N\theta_{r})}{A_{1}\cos\theta_{r}+\sum_{N=2}^{\infty }A_{N}\cos(N\theta_{r})})}{1+\tan\theta_{r}\frac{A_{1}\sin\theta_{r}+\sum_{N=2}^{\infty }A_{N}\sin(N\theta_{r})}{A_{1}\cos\theta_{r}+\sum_{N=2}^{\infty }A_{N}\cos(N\theta_{r})})},$$

(21) $$\mathrm{so}:\Delta \theta =\arctan\left[-\frac{\sum_{N=2}^{\infty }A_{N}\sin((N-1)(\theta_{d}+\Delta \theta ))}{A_{1}+\sum_{N=2}^{\infty }A_{N}\cos((N-1)(\theta_{d}+\Delta \theta ))}\right],$$

According to Equation (21) above, we can see that the harmonic subdivision error consists of different harmonic components, so it is not as simple as the subdivision errors discussed above.

### 2.5. Noise Subdivision Error Analysis

If only the harmonic subdivision error is present, we can introduce the noise components $\delta_{a},\delta_{b}$ into the expression of the subdivision error Δθ and then simplify it as follows:

(22) $$\Delta \theta =\theta_{r}-\arctan\frac{r\sin\theta_{r}+\delta_{a}}{r\cos\theta_{r}+\delta_{b}},$$

(23) $$\mathrm{next}:\tan\Delta \theta =\frac{\tan\theta_{r}-\frac{\sin\theta_{r}+\delta_{a}}{\cos\theta_{r}+\delta_{b}}}{1+\tan\theta_{r}\frac{\sin\theta_{r}+\delta_{a}}{\cos\theta_{r}+\delta_{b}}},$$

(24) $$\begin{array}{l} \mathrm{so}:\Delta \theta =\arctan\left[\frac{\sqrt{\delta_{a}^{2}+\delta_{b}^{2}}\sin(\theta_{d}+\Delta \theta -\lambda )}{r+\sqrt{\delta_{a}^{2}+\delta_{b}^{2}}\cos(\theta_{d}+\Delta \theta -\lambda )}\right],\\ \mathrm{here}:\lambda =\arctan\frac{\delta_{a}}{\delta_{b}}. \end{array}$$

Especially when $\delta_{a}=\delta_{b}=\delta ,$

(25) $$\mathrm{Finally}:\Delta \theta =\arctan\left[\frac{\sqrt{2}\delta \sin(\theta_{d}+\Delta \theta -\frac{\pi }{4})}{r+\sqrt{2}\delta \cos(\theta_{d}+\Delta \theta -\frac{\pi }{4})}\right],$$

According to the equation above, we can use the measured rotary angle $\theta_{d}$ and $\delta_{a},\delta_{b}$ to get the subdivision error Δθ and compensate it.

**Figure 4:**
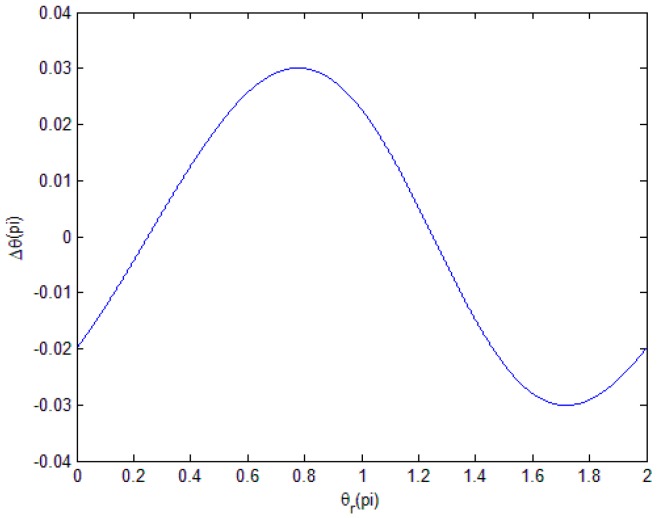
Distribution law of $\Delta \theta_{N}$ in one period of $\theta_{r}$.

Then, we can determine the change of Δθ when $\theta_{r}$ ranges from 0 to 2*π* and obtain the distribution law and period length of the noise subdivision error in Figure 4.

Here, we consider the noise components $\delta_{a}=0.05r,\delta_{b}=0.08r.$

We can see that, in one period of $\theta_{r}$, Δθ is a single period. In almost all spaceborne equipment, the noise should be a minimal component, which can be neglected.

### 2.6. Quantization Subdivision Error Analysis

As we know, the digital code X of a photoelectric angle encoder is uniformly distributed, which means the probability density function of X is as follow [8]:

(26) $$f(x)=\left\{\begin{array}{ll} \frac{1}{b-a} & a<x<b\\ 0 & otherwise \end{array}\right\}.$$

We can obtain the mathematical expectation and variance:

(27) $$E(x)=\int_{a}^{b}x\frac{1}{b-a}dx=\frac{a+b}{2},$$

(28) $$D(x)=E(x^{2})-{\left[E(x)\right]}^{2}=\frac{{\left(b-a\right)}^{2}}{12},$$

When the resolution of the angle code is Q, the quantization subdivision error can be expressed as [8]:

(29) $$\sigma_{x}=\frac{Q}{\sqrt{12}}\approx \frac{Q}{3.46},$$

Here, the quantization subdivision error is the root-mean-square (RMS) error [8], which can be neglected in a standard electric system.

After obtaining the main subdivision error and its expression, we should think of how to compensate it.

All the subdivision errors are listed in the following Table 1:

## 3. Compensation Algorithm and Experiment Set Up

### 3.1. Simulations

**Figure 5:**
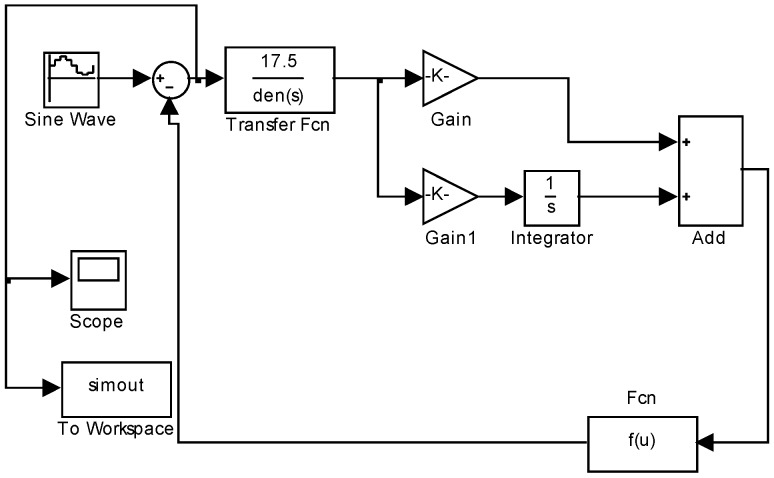
Simulation of the tracking error with subdivision errors.

Once completed the above analysis, we set up a simulation. We assumed three kinds of subdivision error in the system to determine the tracking error before and after compensation. In Figure 5, we considered a sine input x=0.5∗sin(0.05∗t)−10 at the center of minus 10 and added three kinds of subdivision error (amplitude error: dr=0.3r, DC error: a=0.2,b=0, and phase error: φ=10°) in the output to evaluate the tracking error. The transfer function $G(s)=\frac{17.5}{7.842^{-5}s^{2}+0.02143s+1}$ and the controller $C_{PI}(s)=0.1943*\frac{s+62.98}{s}$ were from reference [13]. We determined that the tracking error was 11.15”. Then, we compensated for the amplitude subdivision error, which is the main error, and obtained a tracking error of 6.83”. This means that, after compensating for the main subdivision error, the tracking error decreased of 38.7%. We were not concern about the type of control strategy, because the tracking error would decrease as a result of the measuring error being compensated. In other words, regardless of the type of control strategy, this method can also improve the tracking precision presumably because it compensates the measuring error inside the photoelectric angle encoder.

### 3.2. Compensation Algorithm

**Figure 6:**
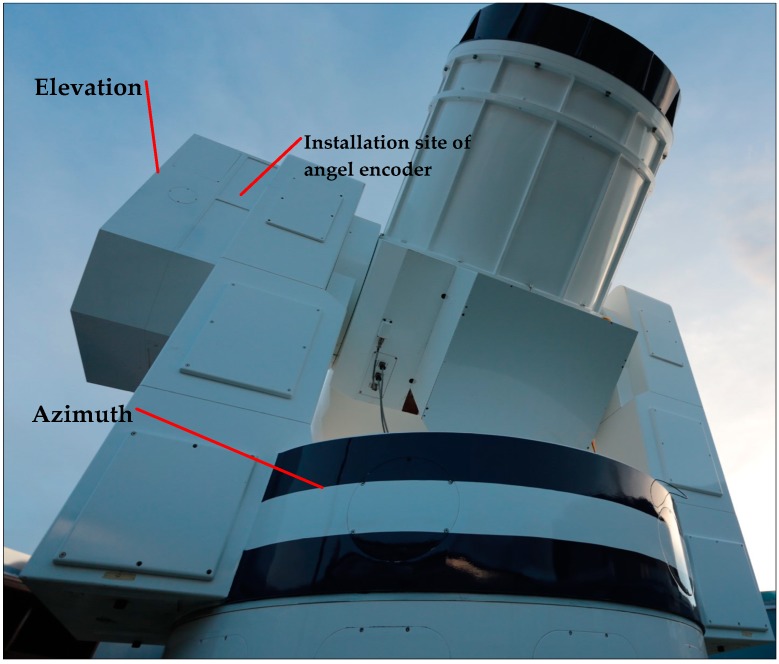
Telescope platform used for the experiment.

**Figure 7:**
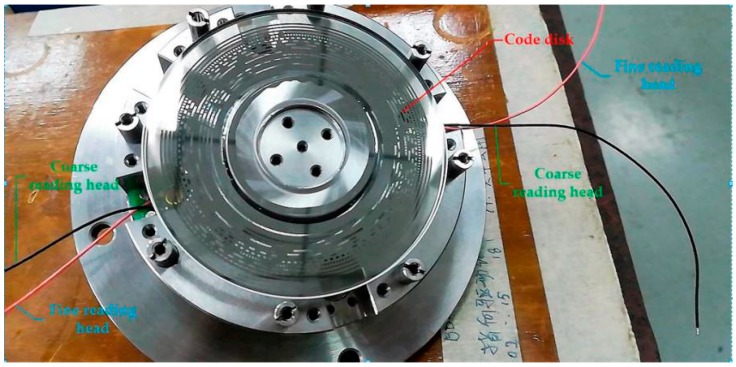
Internal structure of the photoelectric angle encoder.

The experimental telescope platform and photoelectric angle encoder we used are shown in Figure 6 and Figure 7.

To compensate the subdivision error, we should know the structure of the angle encoder and the algorithm to calculate the angle. In Figure 7, we see the code disk of the absolute photoelectric angle encoder, on which there are two coarse-reading heads and two fine-reading heads (indicated in Figure 7 with green and blue lines, respectively). We could read the coarse code and fine code through these reading heads. We removed the processing circuit chip lying on the code disk in order to examine the details of the code disk. The fine code is generated by electronic subdivision, which is processed in the DSP (Digital Signal Processor). The coarse code represents the completed period numbers of subdivision signals *A* and *B* the shaft has rotated. The subdivision of *A* and *B* is described by the fine code, which includes the subdivision errors [14].

**Figure 8:**
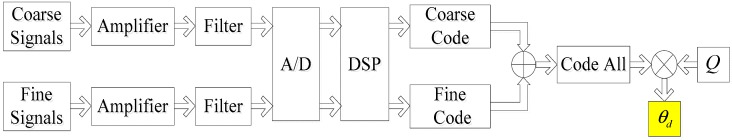
Working process of the angle encoder (including subdivision errors).

The movement of the telescope shaft is transformed to optical signals through the code disk, on which we could obtain the coarse signals and fine signals (electric signals) by the reading heads. In Figure 8, the processing details are discussed. The coarse signals and fine signals were sent into amplifiers and filters to ensure their purity. Afterwards, the analog signals were converted into digital signals by A/D sampling and were then sent into the DSP. After a series of processes, we could determine the measuring angle. However, subdivision errors were present in the angle we obtained. Therefore, a compensation algorithm was required.

**Figure 9:**
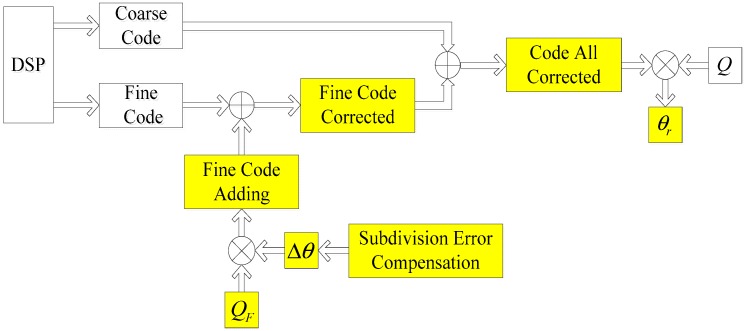
Compensation algorithm for the angle encoder.

In Figure 9, we see the compensation algorithm. First, according to Section 2, we identified the main kind of subdivision error and calculated the compensation angle. In the following Equations, Q is the resolution of the angle encoder, $Q_{C}$ is the resolution represented by the coarse code, $Q_{F}$ is the resolution represented by the fine code. The total bits of the angle encoder are the combination of the coarse code bits and the fine code bits [14]. These parameters are expressed as follows:

(30) $$Q={\left(360/2^{Total\;Bits}\right)}^{{}^{\circ}},$$

(31) $$Q_{C}={\left(360/2^{Coarse\;Bits}\right)}^{{}^{\circ}},$$

(32) $$Q_{F}={\left(360/2^{Fine\;Bits}\right)}^{{}^{\circ}},$$

(33) Total Bits=Coarse Bits+Fine Bits,

(34) $$Code\;All=Coarse\;Code*2^{Fine\;Bits}+Fine\;Code,$$

(35) $$Fine\;Code\;Corrected=Fine\;Code+\Delta \theta *Q_{F},$$

(36) $$Code\;All\;Corrected=Coarse\;Code*2^{Fine\;Bits}+Fine\;Code\;Corrected,$$

(37) $$\theta_{d}=Code\;All*Q,$$

(38) $$\theta_{r}=Code\;All\;Corrected*Q,$$

The processing steps can be described as follows:

Step 1 Obtain the data of coarse code and fine code.
Step 2 According to the analysis of the subdivision errors, identify the main kind of subdivision error and calculate Δθ.
Step 3 Calculate the fine code corrected according to Equation (35).
Step 4 Finally, calculate $\theta_{r}$ through Equations (36) and (38).

Here, we can see that, if we only determined and calculated the subdivision error Δθ, the real rotary angle $\theta_{r}$ is easily obtained. However, it is not obvious how to identify the main kind of subdivision error and calculate it.

### 3.3. Experiment Set Up

**Figure 10:**
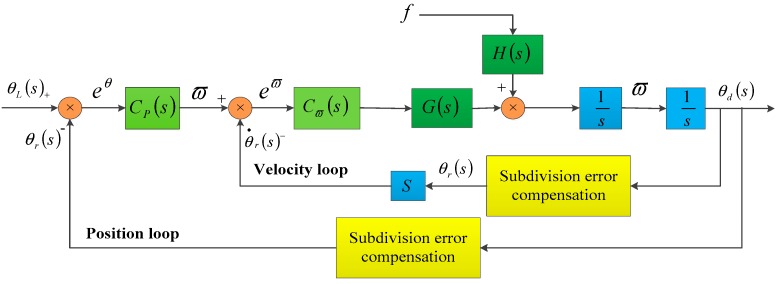
Control system based on classical double-loop structure.

In a real telescope system, the elevation jittering cannot be neglected. Compared with the elevation error, the azimuth error was low, i.e., below the expected value (2″). Therefore, we only consider the elevation as the control target. We used a system with the classical double-loop structure (position loop and velocity loop) [15], as shown in Figure 10.

## 4. Experimental Results

### 4.1. Results before Adding the Compensation Algorithm

**Figure 11:**
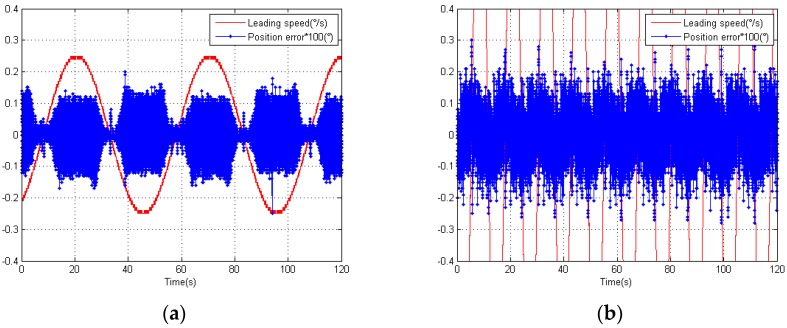
(**a**) Distribution of the position error with leading speed of 0.25°/s; (**b**) Distribution of the position error with leading speed of 1°/s.

The elevation position error, before we applied our compensation algorithm, is shown in Figure 11a,b. The leading speed of the elevation position errors was 0.25°/s and 1°/s. These values were equivalent to sinusoidal guidance whose amplitude and frequency are 1.99°, 0.02 Hz, and 1.99°, 0.08 Hz.

Here, we recorded the leading speed *v*, time *t*, and feedback position $\theta_{d}$ measured by the photoelectric angle encoder. We expressed the leading position as $\theta_{L}=v*t$, so the position error could be expressed as $position\;error=\theta_{L}-\theta_{d}$. Finally, we considered *Error* as the RMS (root mean square) of the position error, $Error={\left(\sum_{i=1}^{N}{\left(position\;error(i)\right)}^{2}/N\right)}^{1/2}$. We could calculate the *Error* of the two kinds of speed: $Error_{v=0.25}=2.31^{\prime \prime }$, $Error_{v=1}=3.01^{\prime \prime }$. Obviously, the elevation error did not satisfy our expectation. Thus, error compensation was necessary.

### 4.2. Results after Applying the Compensation Algorithm

From the analysis in Section 2, we know there are six kinds of subdivision errors that decrease the precision of the angle encoder. However, there must be a main subdivision error out of the six kinds of subdivision errors. To ensure real-time compensation, our algorithm has to compensate only for the main subdivision error on the basis of our analysis above.

The angle encoder we used was 23-bit. More precisely, there were 13 bits for the coarse code and 10 bits for the fine code. According to the expression (31), we determined the resolution of the coarse code, $Q_{C}=360{}^{\circ}/2^{13}=0.0439{}^{\circ}=158.04^{\prime \prime }$. Together with the leading speed *v*, we determined the coarse period time $T_{C}=Q_{C}/v$. Then, we recorded the coarse cycles according to the coarse period time $T_{C}$, and the subdivision error cycles according to Figure 11a,b in one second.

For v=0.25°/s, $T_{C}=\left(0.0439/0.25\right)s=0.176s$. From Figure 11a, we could count the periods. We found that, in one second, there were 1/0.176=5.68 periods for the coarse code and 12 periods for the subdivision error. This means that there were approximately 2.1 periods for the subdivision error in one period of the coarse code.

For v=1°/s, $T_{C}=\left(0.0439/1\right)s=0.0439\;s$. From Figure 11b, we could count the periods. We found that, in one second, there were 1/0.0439=22.78 periods for the coarse code and 46 periods for the subdivision error. This means that there were approximately 2.0 periods for the subdivision error in one period of the coarse code.

We observed that the magnitude subdivision error and phase subdivision error are dual periods that are close to the values we calculated. We also found that the distribution of the error was symmetrical with respect to the center of zero, which suggested that the magnitude subdivision error was the main error.

As we reported, the magnitude subdivision error is:

$$\Delta \theta =\theta_{r}-\arctan\frac{(r-dr)\sin\theta_{r}}{r\cos\theta_{r}},$$

$$\mathrm{so}:\Delta \theta =\arctan(\frac{\sin2\theta_{r}}{\cos2\theta_{r}+\frac{2r}{dr}-1})=\arctan(\frac{\sin(2\theta_{d}+2\Delta \theta )}{\cos(2\theta_{d}+2\Delta \theta )+\frac{2r}{dr}-1}),$$

We defined $\frac{dr}{r}$ and introduced all the required parameters into the TCS, using the compensation algorithm described in Figure 9. We considered *v* = 0.25°/s and *v* = 1°/s after compensation, as indicated below.

**Figure 12:**
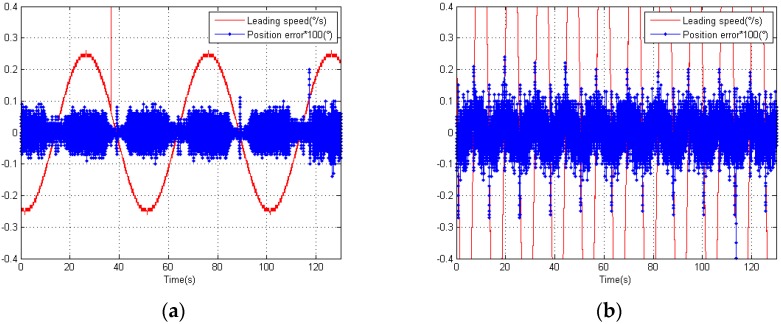
(**a**) Distribution of the compensated position error with a leading speed of 0.25°/s; (**b**) Distribution of the compensated position error with a leading speed of 1°/s.

As shown in Figure 12a,b, the position errors were clearly smaller after compensation. The RMS of the position error after compensation were $Error_{v=0.25}=1.15^{\prime \prime }$ and $Error_{v=1}=1.92^{\prime \prime }$. This means that the errors decreased, respectively, of 50.2% and 36.2%. Therefore, the compensation algorithm considerably increased the precision.

### 4.3. Results after Introducing the Feed-Forward Loop into the TCS

**Figure 13:**
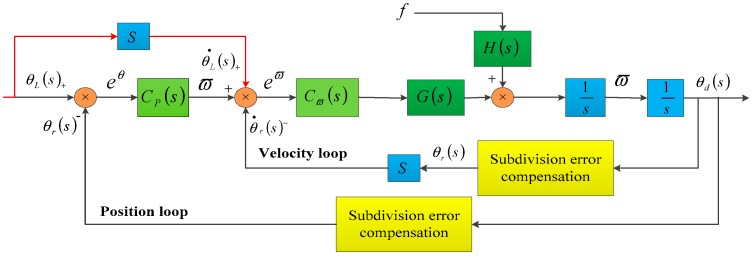
Adding a feed-forward loop into the control system.

In order to further increase the tracking precision, we added a feed-forward loop into the system (see Figure 13). A feed-forward loop can introduce the object properties into the loop and reduce the error.

**Figure 14:**
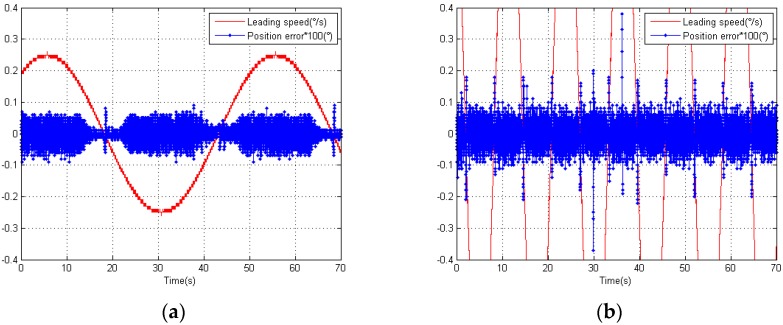
(**a**) Distribution of the compensated position error after adding the feed-forward loop with a leading speed of 0.25°/s; (**b**) Distribution of the compensated position error after adding the feed-forward loop with a leading speed of 1°/s.

After adding the feed-forward loop, the RMS of the position errors were $Error_{v=0.25}=1.05^{\prime \prime }$ and $Error_{v=1}=1.79^{\prime \prime }$. The feed-forward loop further decreased the previously compensated errors of about 9.5% and 6.8%. Because of the characteristics of the feed-forward loop, if the frequency of the error was lower, the error decrease would be greater [16]. The distribution of the error after adding the feed-forward loop was as follows (Figure 14a,b).

## 5. Conclusions

In a real TCS, the elevation jittering phenomenon occurs. We considered the elevation as the control target. After analyzing the period of the position error, we found that magnitude subdivision exists, using the analysis in Section 2. After compensation, the tracking error was decreased by nearly 50.2%, from 2.31” to 1.15” with the leading speed of 0.25°/s, and by 36.2%, from 3.01” to 1.92”, with the leading speed of 1°/s. We observed that, when the speed increased, the tracking error increased, which suggested that when the system worked at a lower speed, the compensation algorithm worked better. We then found that adding a feed-forward loop into the system could further decrease the tracking error, even if only by a few percent points (9.5% and 6.8%). The reason of the small decrease was that the frequency of the error was so high that the feed forward-loop could not run perfectly. Therefore, in the future, we will try to improve the functioning of the feed-forward loop.

The compensation algorithm described above is suitable for complex devices and can provide a real-time compensation without any change of the hardware. It is also adaptable to changing environments, regardless of the control strategy used, and can also improve the tracking precision presumably because it compensate the measuring error inside of the photoelectric angle encoder. Moreover, the compensation method described only requires the position of the shaft that can be easily determined by the photoelectric angle encoder; therefore, its cost is limited, which means it works efficiently.

However, the compensation algorithm has its shortcomings, since it cannot compensate all subdivision errors in the same time. In fact, we observed that other subdivision errors were still present after compensation, and that we only compensated the main error. Therefore, although the compensation algorithm worked well, it still requires improvements. We will work on these improvements in the future to enhance the system’s efficiency.

## Figures and Tables

**Table 1:** Definition of the subdivision errors.

Kind of Errors	Expression of Errors
DC error	$\arctan\left(\frac{\sin\left(\theta_{d}+\Delta \theta -\varphi \right)}{\frac{r}{\sqrt{a^{2}+b^{2}}}+\cos\left(\theta_{d}+\Delta \theta -\varphi \right)}\right)$
Magnitude error	$\arctan\left(\frac{\sin\left(2\theta_{d}+2\Delta \theta \right)}{\cos\left(2\theta_{d}+2\Delta \theta \right)+\frac{2r}{dr}-1}\right)$
Phase error	$\arctan\left(\frac{\tan\theta_{d}}{\cos\varphi_{3}}-\tan\varphi_{3}\right)-\theta_{d}$
Harmonic error	$\arctan\left[-\frac{\sum_{N=2}^{\infty }A_{N}\sin\left(\left(N-1\right)\left(\theta_{d}+\Delta \theta \right)\right)}{A_{1}+\sum_{N=2}^{\infty }A_{N}\cos\left(\left(N-1\right)\left(\theta_{d}+\Delta \theta \right)\right)}\right]$
Noise error	$\arctan\left[\frac{\sqrt{\delta_{a}^{2}+\delta_{b}^{2}}\sin\left(\theta_{d}+\Delta \theta -\lambda \right)}{r+\sqrt{\delta_{a}^{2}+\delta_{b}^{2}}\cos\left(\theta_{d}+\Delta \theta -\lambda \right)}\right]$
Quantization error	×
